# Molecular Alterations of PI3K/Akt/mTOR Pathway: A Therapeutic Target in Endometrial Cancer

**DOI:** 10.1155/2014/709736

**Published:** 2014-01-12

**Authors:** Athanasia Pavlidou, Nikos F. Vlahos

**Affiliations:** ^1^Second Department of Obstetrics and Gynecology, University of Athens Medical School, Aretaieion University Hospital, 76 Vas. Sofias Avenue, 11527 Athens, Greece; ^2^Department of Gynecology and Obstetrics, The Johns Hopkins Hospital School of Medicine, Baltimore, MD 21287, USA

## Abstract

It is well established that the PI3K/Akt/mTOR pathway plays a central role in cell growth and proliferation. It has also been suggested that its deregulation is associated with cancer. Genetic alterations, involving components of this pathway, are often encountered in endometrial cancers. Understanding and identifying the rate-limiting steps of this pathway would be crucial for the development of novel therapies against endometrial cancer. This paper reviews alterations in the PI3K/Akt pathway, which could possibly contribute to the development of endometrial cancer. In addition, potential therapeutic targets of this pathway with emphasis on the mTOR inhibitors are also presented.

## 1. Introduction

Endometrial cancer (EC) is the sixth most common gynecological cancer, causing approximately 74,000 deaths worldwide in 2008 [1]. It is speculated that during 2013, 49,560 new cases and 8,190 deaths will occur in the USA [2]. The majority of EC cases are sporadic but there is a familial predisposition in up to 10% of the cases [3]. According to Bokhman [4], there are two types of endometrial carcinomas: type 1 endometrioid endometrial carcinomas (EECs) represent the majority of sporadic cases of endometrial cancer and account for 70% to 80% of new cases [4]. Type 1 lesions arise in the background of endometrial hyperplasia and overall they are associated with a favorable prognosis. Unopposed estrogen stimulation has been proposed as the main factor associated with the development of this type of carcinomas [5].

On the contrary, type 2 lesions (NEECs) are less common, accounting for 10–20% of endometrial cases [6, 7]. They are not estrogen dependent and arise from a background of atrophic endometrium [4]. They are often high-grade carcinomas with poor prognosis, mainly of the papillary serous and clear-cell type [8].

Accumulating evidence over the past two decades has revealed the role of certain signaling pathways in endometrial carcinogenesis. Better understanding of the underlying oncogenic mechanisms may lead to discovery of novel therapeutic targets and ultimately increase the survival of those patients. One of the most important signaling pathways involved in gynecological carcinogenesis is the PI3K/AKT/mTOR pathway. Amplifications, mutations, and translocations, resulting in aberrant activation of this pathway, occur more frequently than any other pathway in cancer patients [9–13]. The present review will focus on the oncogenic role of mTOR signaling in endometrial tumors as well as potential therapeutic strategies related to this pathway.

## 2. Genetic Alterations of Endometrial Cancer

Apart from the morphologic and clinical features separating type 1 from type 2 ECs, they are further distinguished by specific genetic alterations [14]; EECs are characterized by microsatellite instability (MSI), somatic alterations within the PI3K pathway and the MAPK pathway, and mutations of *CTNNB1* (*β*-catenin) and *ARID1A* (BAF250a) genes. In contrast, NEECs often demonstrate aneuploidy, p53, and PPP2R1A mutations, p16 dysregulation, and significantly less frequent alterations within the PI3K pathway than in EECs.

**Figure 1:**
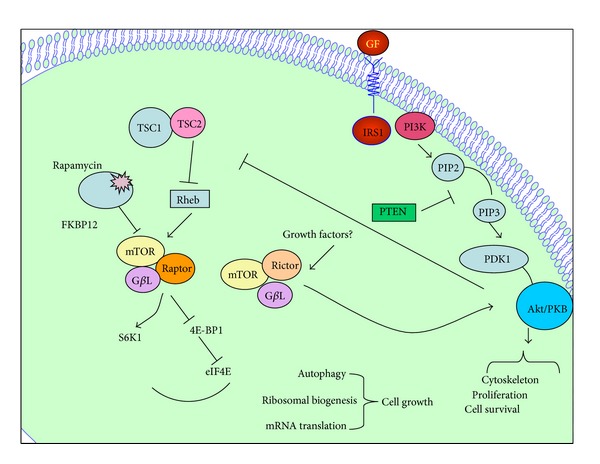
Schematic representation of the PI3K/Akt/mTOR pathway substrates and associated cellular functions. The tumor suppressor protein/lipid PTEN negatively regulates AKT. Following activation, Akt translocates into the cytoplasm and nucleus and phosphorylates TSC2. mTORC1 (mTOR + raptor) and mTORC2 (mTOR + rictor) are two distinct branches of the mTOR pathway. mTORC1 responds to nutrients and growth factors and is regulated by TSC1/2 and Rheb, whereas it is unknown how the mTORC2 complex is regulated. The raptor-mTOR pathway regulates cell growth while rictor-mTOR regulates Akt/PKB to control cell survival, proliferation, and cytoskeleton.

Specifically, the PI3K-Akt signal transduction pathway is the most frequently altered biochemical pathway in EECs; more than 80% of endometrioid endometrial tumors had one or more somatic alterations affecting this pathway [15]. The primary negative regulator of the PI3K pathway is *PTEN*, a well-studied tumor suppressor gene. *PTEN* is located at chromosome 10q23 and encodes for a protein (phosphatase) with tyrosine kinase function. The PTEN product has both lipid and protein phosphatase activity. The lipid phosphatase activity causes cell cycle arrest at the G_2_/S checkpoint and inhibits PI3 phosphorylation by dephosphorylating PIP3 back to PIP2. This decreases intracellular PtdIns levels and affects the downstream Akt signal transduction pathway. The protein phosphatase activity of PTEN product has been found to inhibit cell spreading and migration. Thus, loss of PTEN activity may lead to aberrant cell growth and an escape from apoptosis [16] (Figure 1). PTEN inactivation can be due to either gene mutation, promoter methylation, or protein degradation, which lead to loss of expression, or to a lesser extent, loss of heterozygosity. PTEN alterations are present in 20% of endometrial hyperplasia cases, in 55% of precancerous lesions, in 35–80% of EEC, and in 10% of NEECs [17–22]. *PTEN* mutations are likely an early event in endometrial carcinogenesis, as evidenced by its presence in precancerous lesions.

*PIK3CA*, another gene often mutated in various types of cancer, may also hold a role in the alteration of the phosphatidylinositol 3 kinase (PI3K)/Akt pathway in EC. *PIK3CA* mutations appear in 25–36% of EECs and in 15% of NEECs and they often coincide with PTEN mutations [23–26]. A recent study explored whether mutations of the PI3K pathway, apart from *PI3KCA* and *PTEN*, were present in EC [15]. It has been reported from several groups a mutation rate of *PIK3R1* up to 20%, significantly higher than any other lineage, demonstrating selective targeting in EC [15, 27, 28]. The *PI3KR1* gene encodes for the PI3K regulatory subunit p85a. Several of its mutations are known to phosphorylate AKT, thus activating the downstream signaling pathway [29]. The *PIK3R2* has also been established as a novel cancer gene. The mutation rate for *PIK3R2* has been reported in up to 5% of ECs and several of those mutations have shown to exhibit gain of function [15]. Shoji et al. [30] detected the presence of *AKT1* mutations in 2% of ECs tissue samples. These tumors did not demonstrate any other mutation in *PIK3CA*, *PTEN*, or *K-Ras*. The authors suggested that *AKT1* mutations might be mutually exclusive with other PI3K-AKT activating alterations.

Although high AKT activity is well documented in endometrial adenocarcinomas, very little data exist on the role of the mTOR pathway in this type of cancer. In vivo data on the mTOR cascade components are also lacking. mTOR is the catalytic subunit of two biochemical distinct molecular complexes, mTORC1 and mTORC2. Activation of mTORC1 promotes ribosome biogenesis, increases translation rates and protein synthesis, and inhibits autophagy, thus affecting cell proliferation and cell survival [31]. All these functions are inhibited by rapamycin or rapamycin analogues [32]. Compared with mTORC1, the function of mTORC2 is less well studied, but it is known that mTORC2 activity regulates cytoskeleton organization and promotes activation of AKT (also known as protein kinase B) [33–35].

Darb-Esfahani et al. were among the first to demonstrate activation of p-mTOR and p-4EBP1 in human endometrial adenocarcinomas by immunohistochemistry [36]. Subsequently Shen et al. [37] demonstrated that mTORC2 activity is selectively upregulated in endometrial cancers, as evidenced by the overexpression of nuclear p-mTOR and p-Akt, as well as by the overexpression of VEGF-A isoform and PLD1 in malignant epithelium. The authors suggested that a rapamycin insensitive mTORC2 pathway could play a major role in endometrial tumorigenesis. Targeted therapies blocking the phospholipase D pathway and elements of the mTORC2 pathway could be effective against ECs. In addition, another study by Lu et al. demonstrated that dysregulation of mTOR in primary endometrial carcinomas may be achieved by loss of TSC2 and LKB1 expression (13% and 21%, resp.) [38].

Another important pathway in a variety of human cancers is the Ras/MARK pathway, which interacts with the PI3K pathway through the RAS proteins. This interaction may suggest a cooperation between the two pathways in order to determine functional outcomes. Somatic mutations of the *KRAS* gene are found in 18–28% of EECs [6, 23, 39, 40]. Constitutive activating mutations in K-Ras have been found more frequently in tumors with microsatellite instability (MSI), suggesting that both events may occur simultaneously before clonal expansion [41]. A MSI phenotype is marked by a high frequency of mutations at sites of short nucleotide repeats (microsatellites) within the genome. MSI is the result of unrepaired errors that arise during DNA replication and is detectable in almost 20% of endometrial tumors [42, 43]. In EC, *KRAS* mutations can coexist with mutations in *PIK3CA*, *PIK3R1*, and *PTEN* suggesting that *KRAS* mutations are not functionally redundant with PI3K pathway mutations [15, 25, 29, 44].

## 3. Therapies Targeting the PI3K/Akt/mTOR Pathway

Our knowledge of the molecular pathways involved in endometrial carcinogenesis has led to the development of novel therapeutic agents that target these pathways. Several small-molecule inhibitors and monoclonal antibodies that interfere with mechanisms crucial for cancer development, such as angiogenesis, escaped from apoptosis, cell growth, and metastasis; are now entering clinical trials [45, 46]. Growing evidence suggests that genetic dysregulation of the PI3K-Akt pathway results in the activation of downstream signaling pathways and is responsible for oncogenesis. Components of the Akt pathway may represent potential therapeutic targets [9, 46].

Akt is known to regulate various cellular pathways that promote cell survival, cell proliferation, angiogenesis, and invasion. In view of its antiapoptotic role, Akt over-expression in cancer cells might mediate resistance to radiation or chemotherapy [47]. Inhibition of phosphoinositide-3-kinase (PI3K)/Akt signaling in endometrial carcinomas may be a promising target to enhance the efficacy of anticancer agents such as cisplatin and paclitaxel.

Evidence that the PI3K-Akt pathway can be targeted successfully for clinical use has been provided by studies that used rapamycin to inhibit mTOR, one of the numerous downstream Akt substrates [46, 48]. Akt activity is frequently elevated in ovarian cancer and is closely associated with the upregulation of mTOR signaling [49]. Rapamycin, a highly specific mTOR inhibitor, arrests cells in the G_1_ phase and has shown antitumor activities in vivo as well as in vitro [50, 51]. Currently, mostly in vitro data have supported the antitumor effect of rapamycin and its derivatives in ovarian cancer [49, 52]. Other investigators have shown that rapamycin enhances the effect of cisplatin and carboplatin in ovarian and breast cancer cells with mutant p53 [53, 54]. Similarly, rapamycin potentiates the effect of paclitaxel (inhibition of cellular proliferation, induction of apoptosis, and increased polymerization of tubulin) and cisplatin (inhibition of cell growth, induction of apoptosis, and increased expression of DNA mismatch repair proteins) in endometrial cancer cells [55, 56]. Also, recent clinical studies demonstrated the synergistic effect of rapalogs with chemotherapy in advanced gynecological malignancies and solid tumors [57–59].

Based on the biological rationale of targeting the mTOR pathway, mTOR inhibitors as single agent have entered clinical trials in endometrial cancer [46, 60] (Tables 1 and 2). An orally bioavailable derivative of rapamycin, RAD001 (Everolimus), has been shown to inhibit proliferation of tumor cell growth in vitro and in vivo [61–63]. In addition, Everolimus demonstrated encouraging results in a phase II clinical trial with previously treated patients with progressive or recurrent EEC [64]. In this report, 43% (12 out of 28 patients) of evaluable patients did not demonstrate disease progression at the time of first evaluation. However, the median duration of SD (stable disease) was 4.5 months and eleven patients discontinued treatment either due to toxicity (6 patients) or disease progression (5 patients). Although, mTOR inhibition demonstrated a clinical benefit, the authors believe that disease remission by receiving single agent therapy would be unlikely. Nonetheless, the interruption of a key component in a biologic pathway may be a reasonable approach for disease control.

Another mTOR inhibitor has also entered a phase II trial. Oza et al. [65] evaluated the activity of single-agent Temsirolimus (CC1-779) in women with chemotherapy-naïve or chemotherapy-treated EC. Temsirolimus is a water-soluble ester of rapamycin and is administered by intravenous infusion. In the chemotherapy-naive group, 14% of evaluable patients had a partial response and 69% had stable disease; in the chemotherapy-treated group 4% of patients achieved partial response, while 48% had stable disease. Interestingly, there was no correlation between PTEN loss and other molecular markers of PI3K/Akt/mTOR pathway and clinical response. Temsirolimus is the most advanced of the rapalogs and after a positive phase III [66], the US Food and Drug Administration (FDA) approved it for the first line treatment of poor prognosis patients with advanced RCC.

Colombo et al. studied Ridaforolimus (AP23573), an intravenous mTOR inhibitor, in a phase II trial with recurrent EC. The investigators revealed that 7% of patients had partial response and 26% had stable disease [67]. Mackay et al. focused on endometrial cancer patients who did not receive chemotherapy. In this phase II study (*n* = 34) of Ridaforolimus 7.7% of the patients demonstrated partial response and 53% had stable disease [68].

As expected, however, single-agent treatment with rapamycin and its analogues activates negative feedback mechanisms leading to increased formation of mTORC2 complex, which not only phosphorylates and activates Akt [33, 69] but also promotes eIF4E Ser-209 phosphorylation, favoring its role in the initiation complex [70]. In order to bypass this problem, and induce the maximal inhibition of this pathway, Shoji et al. [71] examined the antitumor effect of combined PI3K/mTOR inhibitor, NVP-BEZ235, and an mTOR inhibitor, RAD001 (Everolimus), in endometrial cancer cells with one or more mutations in *PTEN*, *K-Ras,* and *PIK3CA*. They concluded that a combined PI3K/mTOR inhibition might be more efficacious than mTOR inhibition alone in women with EC [72]. Robust growth suppression of tumor cells with these agents indicates a promising therapeutic strategy. This novel therapeutic agent NVP-BEZ235, which targets both PI3K and mTOR, has been shown to inhibit cell growth of cisplatin-sensitive as well as cisplatin-resistant human ovarian carcinoma cell lines [73, 74]. The effects of this combined PI3K/mTOR inhibitor have been attributed to the induction of cell cycle arrest, apoptosis as well as in its antiangiogenic properties [75–81].

Rapalogs combined to hormonal treatment for gynecological malignancies have also been evaluated in clinical trials [60]. In a phase II randomized clinical trial (*n* = 22) with previously treated patients Temsirolimus (25 mg IV weekly) combined with megestrol acetate (80 mg, twice a day (bid)) for three weeks, alternating in tamoxifen (20 mg bid) for three weeks was compared to Temsirolimus alone [82]. Due to high rate of thromboembolic events in the combination group, the study was terminated prematurely. The interim efficacy analysis demonstrated no significant differences in RR between the two groups.

Another study evaluated the combination of Everolimus (10 mg/day, orally) and letrozole (2.5 mg/day, orally) in patients (*n* = 28) pretreated with chemotherapy [83]. Preliminary data showed a CBR of 43% including four complete responses. In another phase II clinical trial involving patients with advanced endometrial carcinoma [84], Ridaforolimus (*n* = 64) given once daily (40 mg) for five days was compared to either hormonal therapy (*n* = 53) (medroxyprogesterone 200 mg/day or megestrol 60 mg/day) or chemotherapy (*n* = 13). Interim analysis demonstrated a median progression free survival of 3.6 months for Ridaforolimus compared to 1.9 months for those patients treated with hormones,. There was a significant difference in the rate of stable disease between the two groups (35% versus 17%, *P* = 0.02)

Other agents that can affect the PI3K/PTEN/Akt/mTOR signaling pathway have been developed and are currently under investigation in women with endometrial cancer. In a phase II trial of women with recurrent ovarian and endometrial cancer, MKC-1 (EntreMed), an oral cell cycle inhibitor, demonstrated a significant reduction of phospho-Akt [85]. Tanaka et al. examined the PI3K inhibitory activity of a novel agent, CH5132799, in ovarian, endometrial, breast, and prostate cancer cell lines, as well as in xenograft models [86]. CH5132799 is a selective class I PI3K inhibitor with a potent inhibitory activity against PI3K and its mutants. They were able to demonstrate an overall strong antiproliferative activity against the above tumors. In addition, CH5132799 in combination with trastuzumab had a synergistic effect without activation of the negative feedback loop of PI3K/Akt/mTOR signaling [86].

Apart from rapalogs, there is a growing interest in developing inhibitors for the catalytic domain mTOR of the complexes mTORC1 and mTORC2 [46]. A phase I clinical trial for the first oral small molecule mTORC1/mTORC2 inhibitor OSI-027 has been just completed; the study is multicenter and involves three different dosing schedules (ClinicalTrials.gov Identifier: NCT00698243) (Table 2).

## 4. Resistance of mTOR Inhibitors and Future Perspectives

Although clinical studies of rapalogs have shown promising results in renal-cell carcinoma [66, 89, 90], monotherapy with mTOR inhibitors, in other tumors, has shown limited efficacy due to the feedback activation of several survival signaling pathways [91–94]. Resistance to rapalogs could be explained by mechanisms involving the necessity of mTORC1 to directly phosphorylate all the rapamycin-sensitive sites of its substrates, in vivo. One such mechanism, involves structural alteration of mTORC1 after a long-term rapamycin treatment [95]. This may depend on the site of raptor phosphorylation or the dissociation of mLST8 and PRAS40 from mTORC1. Differential phosphorylation by currently unknown kinases or transautophosphorylation may alter the structure of mTORC1. This would specifically recover the phosphorylation of some, but not all, of the substrates.

Another mechanism involves the effect of different binding affinities of mTORC1 substrates [95]. Specifically, it has been observed that S6K1 binds to mTORC1 much less efficiently than 4E-BP1 [96, 97]. In addition, 4E-BP1 T37/46, which are direct mTOR sites in vitro, are not rapamycin sensitive in vivo, whereas T389 on S6K1 is rapamycin-sensitive both in vivo and in vitro. This difference may explain why long-term rapamycin treatment recovers 4E-BP1 but not S6K1 phosphorylation.

Wang et al. suggested a critical role of Akt activation in the development of cell resistance to mTOR inhibitors [98]. In a rapamycin-resistant cell line p-Akt levels increased drastically and remained elevated for a long period after the removal of rapamycin. p-Akt levels returned to normal only after the sensitivity of the rapamycin-resistant cells to mTOR inhibitors was fully restored.

It seems that the survival signaling pathways, PI3K/Akt, MAPK/ERK, and Mnk/eIF4E, are important mediators of resistance to rapalogs. Inhibition of the mTORC1 with a rapalog induces a negative feedback loop activation of the survival pathways, leading to cell resistance to rapalogs [91].

Thus, the combination of rapalogs with other agents (hormonal, chemotherapy, etc.) or the development of novel PI3K and mTOR, combined inhibitors may prevent the feedback loop activation of the survival signaling pathways. Upon that, some mTOR/PI3K dual inhibitors have been developed such as the PI-103 [99] and the NVP-BEZ235 [75], both of which have demonstrated significant antitumor activity.

It has been suggested that another strategy to maximize the clinical effects of mTOR inhibitors would be selection of those patients more likely to respond to mTOR-targeted cancer therapy [46]. Investigation of reliable biomarkers that may predict tumor responses to rapalogs would be an interesting option. Slomovitz et al. [100] in a phase II clinical trial with Everolimus suggested that loss of PTEN in patients with EECs might predict response to the medication. In contrast, Yang et al. using Everolimus in a glioblastoma orthotopic xenograft test panel showed that PTEN loss does not predict response to the treatment [101]. In spite of the increasing interest, currently, there are no potential bio-markers that can aid in selecting patients who can benefit from mTOR inhibitors [102]. Thus, further work is essential in order to understand the biology of mTOR signaling and consequently to develop therapies for all suitable patients on a personalized basis. Additionally, important issue is a therapeutic option that can be economical sustained. Oral rapamycin (Sirolimus), which has been shown to exhibit the same toxicities as its derivatives, it has become widely available after the expiration of the patent in 2013 [103]. The use of the original compound may limit the cost compared with the rest of mTOR inhibitors.

## 5. Conclusions

As discussed above, a link between the mTOR pathway and endometrioid endometrial cancer is clearly evident and most of its upstream and downstream regulators are directly implicated in cancer initiation and progression. Improved understanding of the molecular mechanisms involved in endometrial carcinogenesis has led to the identification and development of molecular target therapies. Encouraging results from in vitro studies and early stage clinical trials of first generation mTOR and PI3K inhibitors in gynecological cancers have recently become available. Moving forward, future phase III studies should evaluate whether rapalogs, either as monotherapy or in combination with other agents, could improve survival in patients who have disease resistant to first-line therapies.

## Figures and Tables

**Table 1:** PI3k/Akt/mTOR inhibitors in preclinical and clinical studies.

Drug	Target kinase	Clinical trial phase	*N*	PR	SD	PFS	References
Preclinical studies							
CH5132799	PI3K						[86]
NVP-BEZ235 + RAD001	PI3K/mTOR						[72]
LY294002 + OBP-801/YM753	PI3K/HDAC						[87]
Published studies and abstracts							
Temsirolimus	mTOR	Phase II	19	7–26%	44–69%	4.3 months	[65, 88]
Ridaforolimus	mTOR	Phase II	45, 34	7%	26–53%	16 weeks	[67, 68]
Everolimus	mTOR	Phase II	35	57%	43%	≥8 weeks	[64]
MKC-1		Phase II	9	55.5%	44.4%	1.8 weeks	[85]
Temsirolimus + megestrol + tamoxifen	mTOR	Phase II	22				[82]
Everolimus + letrozole	mTOR	Phase II	28			≥8 weeks	[83]
Ridaforolimus versus medroxyprogesterone versus chemotherapy	mTOR	Phase II	53		35% versus 17%	3.6 versus 1.9 months	[84]

*N: number of patients; PFS: progression free survival; PR: partial response; SD: stable disease.

**Table 2:** PI3K/Akt/mTOR inhibitors in ongoing trials.

Ongoing trials	Target kinase	Clinical trial phase	*N*	PFS	ClinicalTrials.gov identifier
PF-04691502 + PF-05212384	PI3K/mTOR	Phase II	Recruiting		NCT01420081
XL147	PI3K	Phase II	65	6 months	NCT01013324
MK2206	Akt	Phase II	Recruiting		NCT01307631
Temsirolimus + pegylated liposomal Doxorubicin	mTOR	Phase I	Recruiting		NCT00982631
Ridaforolimus or progestin or chemotherapy	mTOR	Phase II	130		NCT00739830
Ridaforolimus + paclitaxel + carboplatin	mTOR	Phase I	Recruiting		NCT01256268
Temsirolimus + bevacizumab	mTOR + VEGF	Phase II	Recruiting		NCT01010126
OSI-027	mTORC1 + mTORC2	Phase II	128		NCT00698243

*N: number of patients; PFS: progression free survival.
